# Facile Bio-Fabrication of Ag-Cu-Co Trimetallic Nanoparticles and Its Fungicidal Activity against *Candida auris*

**DOI:** 10.3390/jof7010062

**Published:** 2021-01-18

**Authors:** Majid Rasool Kamli, Vartika Srivastava, Nahid H. Hajrah, Jamal S. M. Sabir, Khalid Rehman Hakeem, Aijaz Ahmad, Maqsood Ahmad Malik

**Affiliations:** 1 Department of Biological Sciences, Faculty of Science, King Abdulaziz University, P.O. Box 80203, Jeddah 21589, Saudi Arabia; mkamli@kau.edu.sa (M.R.K.); nhajrah260@gmail.com (N.H.H.); jsabir2622@gmail.com (J.S.M.S.); kur.hakeem@gmail.com (K.R.H.); 2 Center of excellence in Bionanoscience Research, King Abdulaziz University, Jeddah 21589, Saudi Arabia; 3 Clinical Microbiology and Infectious Diseases, Faculty of Health Sciences, School of Pathology, University of the Witwatersrand, Johannesburg 2193, South Africa; vartika.srivastava@wits.ac.za (V.S.); Aijaz.Ahmad@wits.ac.za (A.A.); 4 Infection Control Unit, Charlotte Maxeke Johannesburg Academic Hospital, National Health Laboratory Service, Johannesburg 2193, South Africa; 5 Chemistry Department, Faculty of Science, King Abdulaziz University, P.O. Box 80203, Jeddah 21589, Saudi Arabia

**Keywords:** nanoparticles, green synthesis, *Candida auris*, apoptosis, G2/M arrest, cell cycle

## Abstract

*Candida auris* is an emergent multidrug-resistant pathogen that can lead to severe bloodstream infections associated with high mortality rates, especially in hospitalized individuals suffering from serious medical problems. As *Candida auris* is often multidrug-resistant, there is a persistent demand for new antimycotic drugs with novel antifungal action mechanisms. Here, we reported the facile, one-pot, one-step biosynthesis of biologically active Ag-Cu-Co trimetallic nanoparticles using the aqueous extract of *Salvia officinalis* rich in polyphenols and flavonoids. These medicinally important phytochemicals act as a reducing agent and stabilize/capping in the nanoparticles’ fabrication process. Fourier Transform-Infrared, Scanning electron microscopy, Transmission Electron Microscopy, Energy dispersive X-Ray, X-ray powder diffraction and Thermogravimetric analysis (TGA) measurements were used to classify the as-synthesized nanoparticles. Moreover, we evaluated the antifungal mechanism of as-synthesized nanoparticles against different clinical isolates of *C. auris*. The minimum inhibitory concentrations and minimum fungicidal concentrations ranged from 0.39–0.78 μg/mL and 0.78–1.56 μg/mL. Cell count and viability assay further validated the fungicidal potential of Ag-Cu-Co trimetallic nanoparticles. The comprehensive analysis showed that these trimetallic nanoparticles could induce apoptosis and G2/M phase cell cycle arrest in *C. auris*. Furthermore, Ag-Cu-Co trimetallic nanoparticles exhibit enhanced antimicrobial properties compared to their monometallic counterparts attributed to the synergistic effect of Ag, Cu and Co present in the as-synthesized nanoparticles. Therefore, the present study suggests that the Ag-Cu-Co trimetallic nanoparticles hold the capacity to be a lead for antifungal drug development against *C. auris* infections.

## 1. Introduction

With the advent of HIV and other immunocompromising diseases and modern-day surgeries, an increase in *Candida* infections has been reported. These infections have been recognized as a global threat, further amplified by the spread of multidrug-resistant *Candida auris. Candida auris*, an emerging threat to immunocompetent and immunocompromised patients worldwide, is often resistant to available antifungal drugs [1,2,3]. This species is generally unresponsive to fluconazole and often resistant to amphotericin B and therefore, echinocandins are the first-line drugs for treating *C. auris* infections [4]. Therefore, tackling this pan-resistant *Candida* species with limited drug classes is very difficult, which advocates the indispensable need for new antimycotic molecules with novel and alternative modes of action against *C. auris*. Nanoscience can create a safer, clean and valuable living ecosystem where, through the use of relatively greener nanotechnologies, industrial chemicals and harmful pollutants can be remedied [5,6]. The development of diverse nanomaterials with exceptional and unique properties is a pioneering way of solving challenges/problems for renewable energies, human health, effective drug provision and sophisticated treatments. Still, extensive exploration is needed in this area. Nanoparticles and nano-scaled materials, particularly metallic nanoparticles, contain new and exceptional physicochemical and biological properties [7,8,9,10]. The practical and promising antimicrobial ability of trimetallic nanoparticles was more than the mono and bi-metallic equivalents. For example, CuO-NiO-ZnO mixed nanoparticles with metal oxide exhibited good antibacterial activity and detrimental effects on *E. coli* and *S. Aureus* bacterial strains and nanoparticles (NPs) affixed to the bacterial cell wall exterminate the bacterial cells [11]. The green-synthesized trimetallic Au-Pt-Ag NPs demonstrated the potential for anti-biofilm and antimicrobial activities against *S. aureus*, *E. Coli*, *Enterococcus faecium*, *Candida albicans* and *Enterococcus faecalis*, [12]. Metal nanoparticles are divided into monometallic, bimetallic and trimetallic nanoparticles/oxides according to the number of metal precursors involved in the nanoparticle formation. Among these materials, multi-metallic nanoparticles show higher catalytic activity, enhanced antimicrobial action, diverse morphology, highly selective and sensitive detection, increased efficiency of drug encapsulation, good stability and chemical transformation compared to monometallic nanoparticles [13,14,15,16,17,18,19]. The synergistic or multi-functional effect of the two or three metals present in the multi-metallic nanoparticles is attributable to these promising properties. The synergism plays a significant role in the enhanced antimicrobial efficiencies of multi-metallic nanoparticles surpassing monometallic counterparts because of the integration of different metals [14,15,16,17,18,19,20]. In recent times, a detailed study has been carried out on the synergistic effect of bimetallic nanoparticles against bacteria and yeast species with characteristic Minimum Inhibitory Concentration (MIC) values [21,22,23].

Moreover, the possibilities for the various morphologies and configurations of multi-metallic nanoparticles, such as core-shell, mixed configurations, sub-cluster separated and multi-shell, are increased because of the presence of multi-metals in these materials [24]. Monometallic, bimetallic and multi-metallic nanoparticles are often synthesized through chemical or physical methods [25,26,27,28,29,30,31,32,33,34,35]. However, there are many benefits to using green approaches, such as flexibility, environmental friendliness, cost-effectiveness and protection [36,37,38,39,40,41,42,43]. Three key factors to be carefully taken care of in the green synthesis method are the choice of green sources for solvent, reducing agent and stabilizing/capping agent [38,39,40,41,42,43].

In this study, novel trimetallic nanoparticles of Ag-Cu-Co were synthesized and tested for their antifungal activity against different *C. auris* isolates. To further understand the in-depth antifungal mode of action of Ag-Cu-Co trimetallic nanoparticles, induction of apoptosis and cell cycle arrest studies in *C. auris* were carried out. Apoptosis is a highly controlled mechanism in yeast and can be controlled by various extrinsic and intrinsic factors. The manipulation in apoptosis by external insults can be utilized to develop novel antifungal drugs, which do not rely on the classical antifungal drug targets [44]. Several biochemical and morphological changes can differentiate programmed cell death or apoptosis from accidental cell death or necrosis. Different hallmarks such as externalization of phosphatidylserine, mitochondrial depolarization and *Cytochrome c* oxidase activity were studied to investigate apoptosis in yeast cells [45]. Furthermore, the cell cycle is an essential nuclear event in the process of cellular proliferation. Only cells with intact DNA can cross the cell cycle checkpoints and thereby trapping the mutated DNA cells in different phases. The potential of therapeutic agents to target cell cycle arrests in different phases can be utilized to develop novel therapeutic agents with varying mechanisms of antifungal action. Therefore, nanoparticles with potent anti-candidal activity and inducing apoptosis and cell cycle arrest in *C. auris* can be regarded as powerful lead anti-*Candida* agents and efficient future therapeutic options.

## 2. Materials and Methods

### 2.1. Materials

Deionized water was used as a solvent in the preparation of extract and nanoparticles. Metal precursors salts including copper(II) nitrate trihydrate (Cu(NO_3_)_2_. 3H_2_O, 99%) Molecular weight = 241.60 g/mol, cobalt(II) nitrate hexahydrate (Co(NO_3_)_2_. 6H_2_O, 99%) Molecular weight = 291.03 g/mol, silver nitrate (AgNO_3_, 99%) Molecular Weight = 169.87 were acquired from Sigma-Aldrich. Solvents were also procured from Sigma-Aldrich with a purity of 99%. The purchased chemicals were of analytical grade reagents and used as received. The *Salvia officinalis* leaves were purchased from the local market in Jeddah, Saudi Arabia. Nanoparticles synthesis experiments were performed in Ultrapure water (Millipore, 18.2 MΩ cm).

### 2.2. Preparation of the Extract

The leaves of Salvia officinalis, also known as common sage, were first washed with tap water, followed by several piles of washing using distilled water to remove all impurities and dust particles from the surface of leaves. The rinsed leaves were dried at room temperature and grounded into a powder using mortar and pestle. From this dried Salvia officinalis powder, 5 g was poured into a 250 mL beaker containing 200 mL double distilled water. The mixture was heated for 1 h at 50 °C and kept unfiltered overnight at room temperature. The cooled plant extract was filtered with Whatman filter paper No. 1 using a vacuum pump and extract was stored at 4 °C in dark bottles for further use in nanoparticle preparation.

### 2.3. Preparation and Characterization of Ag-Cu-Co Trimetallic Nanoparticles

In a typical reaction procedure, trimetallic nanoparticles of Ag-Cu-Co were synthesized by taking equal volumes of metal precursor solution of Ag (20 mL, 0.01 mol dm^−3^), Cu (20 mL, 0.01 mol dm^−3^) and Co (20 mL, 0.01 mol dm^−3^) in a beaker and stirred on a magnetic stirrer plate at 40 °C to achieve a homogeneous mixture. To this reaction mixture of metal precursors, 30 mL of the aqueous extract of *Salvia officinalis* was added under continuous stirring at 40 °C. Finally, the reaction mixture’s color changed from light purple to dark greenish, indicating the nanoparticles’ formation. The dark greenish colored solution was centrifuged for 30 min at 20,000 rpm to collect the solid material for further characterization and confirmation of the Ag-Cu-Co trimetallic nanoparticle formation. These acquired phytochemical mediated Ag-Cu-Co trimetallic nanoparticles were washed several times with ultrapure distilled water and finally washed with ethanol to remove impurities from the surface of as-prepared materials. Yet, the acquired solid greenish solid material was dried in an oven at 100 °C for 12 h and store in a dry place under vacuum condition for further spectroscopic and microscopic characterization and applications as an antifungal agent. The biosynthesized Ag-Cu-Co trimetallic nanoparticles were characterized using Fourier transform-infrared (FTIR) spectroscopy (Bruker FTIR (Model: ALPHA II), range of 400–4000 cm^−1^), X-ray diffraction (XRD) spectroscopy (Bruker AXSD8 advance) with Cu Ka (k = 1.5418 Å), Scanning electron microscopy (SEM) (FEG-SEM: Zeiss 540 ultra), transmission electron microscopy (TEM) (JEOL JEM-2100F Field emission microscopy) equipped with energy-dispersive X-ray (EDX) spectroscopy and Thermogravimetric analysis (TGA) measurements (Perkin Elmer, Pyris Diamond) at a heating rate of 10 °C/min under the nitrogen atmosphere.

### 2.4. Biological Assays

In the present study, *C. auris* strains (*n* = 25) procured from the National Institute of Communicable Diseases (NICD), South Africa and preserved in the department were used. All these isolates were previously tested for drug susceptibility against azoles, polyenes and echinocandins and, based on those results, have been categorized as drug-sensitive or resistant [4]. An ethics approval for using the clinical strains for experimental purposes was granted by Wits Human Research Ethics Committee (M140159).

### 2.5. Antifungal Activity of Ag-Cu-Co Trimetallic Nanoparticle

The in vitro antifungal activity of Ag-Cu-Co trimetallic nanoparticles and three known antifungal drugs against 25 clinical strains of *C. auris* was performed by broth microdilution assay as per Clinical and Laboratory Standards Institute guidelines (CLSI 2008). The concentration range of Ag-Cu-Co trimetallic nanoparticles, amphotericin B, fluconazole and caspofungin used for this assay was 25 − 0.04, 16 − 0.031, 1000 − 3.91 and 16 − 0.031 µg/mL, respectively. Furthermore, in every experiment, negative (1% DMSO), culture and media controls were included. MIC values were recorded based on visual observations.

The minimum fungicidal concentration (MFC) values for Ag-Cu-Co trimetallic nanoparticles were evaluated by sub-culturing 10 µL from wells showing no turbidity on SDA plates. Results were recorded after incubating the plates 37 °C for 24 h.

### 2.6. Cell Count and Viability

The fungicidal activity of Ag-Cu-Co trimetallic nanoparticles was quantified by cell count and viability assay using Muse^TM^ Count and Viability assay kit, following the manufacturer’s instructions. Briefly, *C. auris* MRL6057 cells were grown and exposed to ½ MIC, MIC and MFC values of nanoparticles and incubated at 37 °C for 4 h. After that, exposed yeast cells were washed and an aliquot of 20 µL was mixed with 380 µL of Count & Viability reagent, followed by 5 min incubation at room temperature. Muse^TM^ Cell Analyzer examined the cell count and viability of treated and untreated yeast cells. The yeast cells exposed to H_2_O_2_ (10 mM; Merck, Germany) were taken as a positive control, whereas healthy untreated cells were considered negative control.

### 2.7. Apoptotic Studies

#### 2.7.1. Protoplast Preparation

According to the method described previously, protoplasts were prepared from *C. auris* MRL6057 cells [46]. Cells in a mid-log phase were exposed to ½ MIC, MIC and MFC values of nanoparticles for 4 h at 37 °C and were treated with protoplast buffers 1, 2 and 3. Finally, protoplasts were precipitated at 1500 rpm for 5 min and were mixed gently with sterile PBS and stored at 4 °C for shorter durations until further use.

#### 2.7.2. Effect on *C. auris* Mitochondrial Membrane Potential (Δψm)

The effect of Ag-Cu-Co trimetallic nanoparticles on *C. auris* MRL6057 Δψm was evaluated by JC-10 mitochondrial membrane potential assay kit (Abcam, UK), following the manufacturer’s instruction. *C. auris* protoplast suspension (90 μL) was mixed with JC-10 dye-loading (50 µL) and loaded in predefined wells of clear bottom-black walled 96-well microtiter plates (Thermo Fisher Scientific, Germany), incubated in the dark for an hour at room temperature. Post-incubation, the buffer-B (50 µL) supplied in the kit was added to all the wells and the plate was centrifuged at 800 rpm for 2 min. A SpectraMax iD3 multi-mode microplate reader (Molecular Devices, USA) was used to record Ex/Em = 490/530 nm and 540/590 nm. The green fluorescence (mentioned as X) was recorded at Ex/Em = 490/530 nm whereas, red fluorescence (mentioned as Y) was recorded at Ex/Em = 540/590 nm. To evaluate the difference in mitochondrial membrane potential, the ratio of JC-10 aggregates (Y mean) and JC-10 monomeric (X mean) forms were calculated. A decreased ratio (Y mean/X mean) confirmed the mitochondrial membrane’s depolarization due to exposure to the nanoparticles. *C. auris* cells exposed to H_2_O_2_ (10 mM) were taken as a positive control, whereas healthy untreated cells were considered as a negative control.

#### 2.7.3. Effect on *Cytochrome c* Discharge

The discharge of *Cytochrome c* after exposure to Ag-Cu-Co trimetallic nanoparticles was studied, as described by Yun and Lee [47]. Briefly, 0.5 McFarland yeast cells were exposed to the nanoparticles at varied concentrations (½ MIC, MIC and MFC) for 4 h at 37 °C, with shaking. Next, cells were precipitated, washed with fresh PBS and homogenized in buffer A [0.002 M, EDTA (Merck, Germany); 0.001 M, Phenylmethylsulfonyl fluoride (Roche Diagnostics, Germany); 0.05 M, Tris base (pH 7.5)]. After homogenization, cells were precipitated at 4000 rpm for 10 min and the supernatant was secured in separate tubes. The supernatant was again centrifuged for 45 min at 50,000 rpm. The supernatant was confirmed in a separate tube and used to evaluate the cytosolic *Cytochrome c* level. The remaining pellet was dissolved in buffer B [EDTA, 0.002 M; Tris base, 0.05 M; pH 5.0] and the level of mitochondrial *Cytochrome c* level was estimated. The *Cytochrome c*, both cytosolic and mitochondrial, were reduced by adding ascorbic acid (500 mg/mL; Sigma Aldrich Co., St. Louis, MO, USA) and further analyzed by UV-1800 SHIMADZU spectrophotometer at 550 nm.

#### 2.7.4. Annexin V-FITC/PI Staining

Phosphatidylserine (PS) externalization in the cell membrane is an indication of the initial phase of apoptosis. Detection of transfer of PS from the inner side to the outer side of the cell membrane was done using Apoptosis Detection Kit I (BD Biosciences, CA, USA) by following instructions provided by the manufacturer. BD D LSRFortessa Flow cytometer (Becton Dickinson, NJ, USA) analyzed the treated and untreated protoplasts and results were analyzed using FlowJo_V10 software. Quadrant 1, quadrant 2, quadrant 3 and quadrant 4 respectively represent necrosis (Annexin V^−^/PI^+^), late apoptosis (Annexin V^+^/PI^+^), early apoptosis (Annexin V^+^/PI^−^) and live cells (Annexin V^−^/PI^−^). In every set of experiments, both negative and positive controls were included.

#### 2.7.5. Cell Cycle Arrest

Muse^TM^ Cell Analyzer was used to study the consequences of Ag-Cu-Co trimetallic nanoparticles on the cell cycle. For this purpose, Muse™ Cell Cycle kit was used and instructions, as mentioned by the manufacturer, were followed. Briefly, *C. auris* MRL6057 cells were propagated till mid-log phase, later precipitated at 3000 rpm for 4 min, suspended in SDB (0.5 McFarland) and exposed to different concentrations of nanoparticles (½ MIC, MIC and MFC) for 4 h. After that, cells were washed with sterile PBS and the pellet was secured and fixed in 70% chilled ethanol (1 mL; Sigma Aldrich Co., St. Louis, MO, USA). The fixed cells (200 µL) were transferred in a separate tube, centrifuged and washed with fresh PBS. Muse™ Cell Cycle reagent (200 µL) was added to the tube containing fixed cells, incubated in the dark for 30 min. Similar treatments were given to positive and negative controls. Post-incubation samples were examined by Muse^TM^ Cell Analyzer and results were recorded.

### 2.8. Cytotoxicity

The cytotoxic effect of Ag-Cu-Co trimetallic nanoparticles (½ MIC, MIC and MFC) was evaluated in terms of percent hemolysis, using horse red blood cells (NHLS, South Africa) as described by Lone and his coworkers [46]. The positive control was maintained using Triton X-100 (1%), whereas fresh PBS was used as a negative control. The percent hemolysis was calculated using the Formula (1) mentioned below:

(1) $$\%\;Haemolysis=\frac{\left[\left(A450\;of\;treated\;sample\right)-\left(A450\;of\;negative\;control\right)\right]}{\left[\left(A450\;of\;positive\;control\right)-\left(A450\;of\;negative\;control\right)\right]}\times 100$$

### 2.9. Statistics

The graphs were statistically analyzed by GraphPad Prism software, version 8.0.1, using a Two-way ANOVA test. Experiments were executed individually in triplicates at three different time intervals and the results were presented as means ± standard error. The statistical analysis with *p*-values ≤ 0.05 was considered significant.

## 3. Results and Discussion

### 3.1. Phytogenic Synthesis of Ag-Cu-Co Trimetallic Nanoparticles

**Figure 1:**
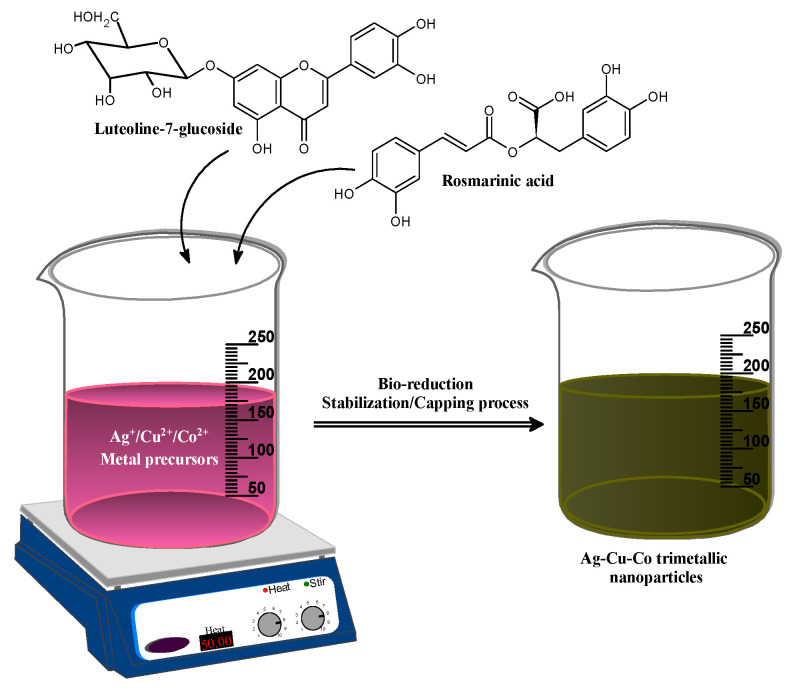
The Bio-fabrication mechanism of *Salvia officinalis* assisted Ag-Cu-Co trimetallic nanoparticles.

The Ag-Cu-Co nanoparticles were synthesized by a simple, one-pot, seedless in-situ method treating a solution of metal precursors including Ag, Cu and Co with aqueous extract of *Salvia officinalis.* Since we have not used any external reducing agent, it is logical to assume that the phytochemical constituents present in the extract performed the reduction Ag^+^ to (Silver nanoparticles) AgNPs, Cu^2+^ to (Copper nanoparticles) CuNPs and Co^2+^ to (Cobalt nanoparticles) CuNPs according to their corresponding reduction potentials (Ag^+^ + ē ⟶ Ag⁰ (0.88 V), Cu^2+^ + 2ē ⟶ Cu (0.34 V), Co^2+^ + 2ē ⟶ Co (−0.28 V)). On addition of the aqueous extract of *Salvia officinalis* to the metal precursor solution, the colors change take place from light purple to dark greenish color, which provides the initial indication of Ag-Cu-Co nanoparticle formation (Figure 1). The use of phytochemicals as bio-reducing and stabilizing/capping agents results in improved fabrication of nanoparticles with high stability and an improved monodispersed nature without the requirement for any complex process during the phytonanofabrication of nanoparticles. The presence of flavonoids, including rosmarinic acid and luteolin-7-glucoside, significantly reduces the potential for Ag-Cu-Co’s synthesizing metal nanoparticles [38,48,49]. Hence, we believe that the rosmarinic acid and luteolin-7-glucoside gallic acid have acted as bio-reducing and capping/stabilizing agents in our case [39]. However, the possibility of other minor constituents cannot be completely ruled out. Elucidation of the actual mechanism and biochemical pathways leading to metal nanoparticles biosynthesis is necessary to develop a rational approach in this field. Therefore, research on the underlying molecular mechanism is essential to control the metal nanoparticles’ size, shape and crystallinity. However, the exact mechanism behind the phytosynthesis of metal nanoparticles is unknown and more detailed studies are needed [50].

**Figure 2:**
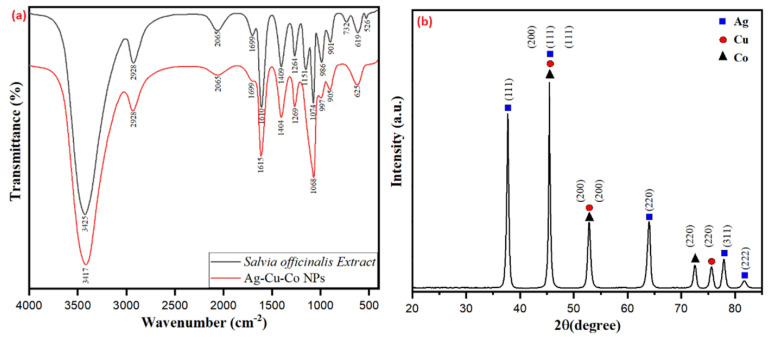
(**a**) Fourier transform infrared (FTIR) spectra of *Salvia officinalis* assisted Ag-Cu-Co trimetallic nanoparticles. (**b**) The X-ray diffraction (XRD) patterns of *Salvia officinalis* assisted Ag-Cu-Co trimetallic nanoparticles.

The *Salvia officinalis* derived Ag-Cu-Co trimetallic nanoparticles were characterized for the possible role of bioactive molecules present in the aqueous extract, especially the engaged functional groups responsible for bio-reduction stabilization/capping of the nanoparticles. The FTIR spectrum of *Salvia officinalis* shows strong peaks at 3425 cm^−2^, 2928 cm^−1^, 1699 cm^−1^, 1610 cm^−2^, 1409 cm^−2^, 1263–1074 cm^−2^ and 986–732 cm^−2^ assigned to strong and broad -OH stretching, medium C-H stretching, C=O stretching, strong C=C stretching, medium -OH bending, strong C-O stretching and strong C=C bending, respectively (Figure 2a). The FTIR analysis of biosynthesized Ag-Cu-Co trimetallic nanoparticles clearly shows the peaks present in *Salvia officinalis* extract, suggesting the apparent involvement of these phytochemicals in bio-fabrication and capping/stabilizing of as-synthesized nanoparticles. However, it was observed that a significant shift in the major peaks takes place (3425 cm^−1^ to 3417 cm^−1^), (1610 cm^−1^ to 1615 cm^−1^), (1409 cm^−1^ to 1404 cm^−1^), (1264 cm^−1^ to 1269 cm^−1^) and (1074 cm^−1^ to 1068 cm^−1^), which suggests the involvement of coordinative interaction of these groups with the metal precursors and forms a capping layer over the surface of the bio-reduced metal nanoparticles (Figure 2a). This indicated the possible involvement of the hydroxyl (-OH) groups of biomolecules present in the aqueous extract of *Salvia officinalis* leaves for bio-reduction and capping/stabilization of the metal nanoparticles. The involvement of phytochemicals of *Salvia officinalis* leaf extract in surface capping and stabilization of Ag-Cu-Co trimetallic nanoparticles is apparent from the FTIR spectral results depicted in Figure 2a. Compared to Ag-Cu-Co trimetallic nanoparticles, a few bands present in the extract’s FTIR spectra exhibit varying intensity and appreciable shifts (Figure 2a).

The preparation of Ag-Cu-Co trimetallic nanoparticles was furthermore confirmed by investigating the XRD pattern. The XRD pattern provides a perception concerning the crystallinity, purity and size of the nanoparticles. Figure 2b represents the XRD spectrum of biosynthesized Ag-Cu-Co trimetallic nanoparticles. Well-defined diffraction patterns of Ag-Cu-Co nanoparticles were observed, indicating that the as-synthesized nanoparticles are perfectly crystalline. The diffraction peaks at 2θ of 37.74°, 45.44°, 63.98°, 77.91° and 81.73° are attributed to the Miller-Bravais indices of (111), (200), (220), (311) and (222) planes correspond well with the fcc crystal structure of Ag (JCPDS 01-087-0720). The diffraction peaks that appeared at 2θ value of 45.44°, 52.87° and 75.60° corresponding to (111), (200) and (220) crystallographic planes of fcc structure of metallic Cu (JCPDS 04-0836). Similarly, the characteristic diffraction peaks of Co-located at 45.44°, 52.87° and 72.54° are ascribed to the Miller-Bravais indices of (111), (200) and (220) corresponds well with Co (JCPDS 15-0806) planes. Ag, Cu and Co diffraction peaks were observed from our results, indicating that the as-synthesized trimetallic nanoparticles consist of Ag, Cu and Co phases. It is significant to note that no impurity or any oxide peak was observed, indicating the formation of oxide-free Ag-Cu-Co trimetallic nanoparticles. The crystalline size and the nanoparticle identification in biosynthesized Ag-Cu-Co trimetallic nanoparticles are given in Table 1. The crystallite size of the Ag-Cu-Co trimetallic nanoparticles was found to be 17.03 nm calculated by using Scherrer’s Equation (2):

(2) $$d=\frac{K\lambda }{\beta \cos\theta },$$

where *d* corresponds to the particle size, K is the shape-dependent Scherrer’s constant, λ is the wavelength of radiation, β is the full peak width at half-maximum (FWHM) of the peak and θ is the Bragg diffraction angle [10].

**Figure 3:**
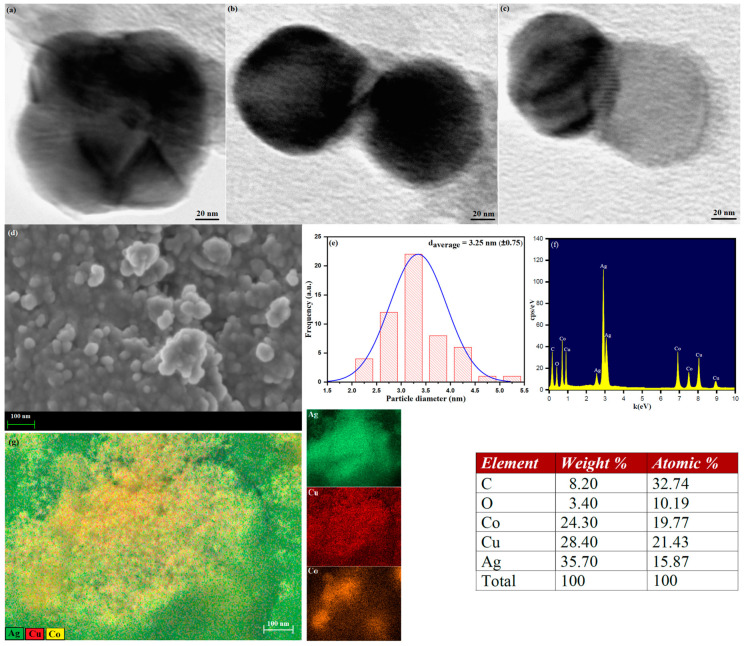
Transmission electron microscopy (TEM) images (**a**–**c**) single particle of Ag-Cu-Co trimetallic nanoparticles, (**d**) Scanning electron microscopy (SEM) image of Ag-Cu-Co trimetallic nanoparticles, (**e**) Particle size distribution histogram, (**f**) Energy-dispersive X-ray spectroscopy (EDX) elemental data of Ag-Cu-Co trimetallic nanoparticles and (**g**) Energy dispersive spectroscopy (EDS) elemental mapping images for Ag-Cu-Co trimetallic nanoparticles.

The phytochemical capped Ag-Cu-Co trimetallic nanoparticles’ surface morphology was further analyzed by transmission electron microscopy (TEM) and scanning electron microscopy (SEM). The single-particle TEM images of Ag-Cu-Co trimetallic nanoparticles are shown in Figure 3a–c. The TEM micrographs revealed that the as-synthesized trimetallic nanoparticles are well-dispersed with spherical shape and an average particle size of 3.25 nm. Figure 3d represents the SEM image of biosynthesized Ag-Cu-Co trimetallic nanoparticles. From the surface analysis, it is evident that the particles are spherical, monodispersed without agglomeration (Figure 3d). Using a particle size analyzer (ImageJ software), the particle size distribution histogram and average particle size (3.25 nm, ±0.75) of Ag-Cu-Co nanoparticles were determined by plotting the diameter histogram and the average particle size was calculated using Gaussian fit (Figure 3e). Energy-dispersive X-ray microanalysis (EDX) is one of the sensitive methods for surface analysis to determine the Ag-Cu-Co trimetallic nanoparticles’ elemental composition. The EDX spectra of Ag-Cu-Co nanoparticles shown in Figure 3f clearly show the presence of elemental peaks of Ag, Cu and Co with a weight % ratio of 35.70, 28.40 and 24.30, respectively, indicate the formation of Ag-Cu-Co nanoparticles in the ration of 3:2:1. Thus, the elemental peaks of Ag, Cu and Co conform to the formation of trimetallic nanostructures of Ag-Cu-Co. The existence of weak C and O signals may be attributable to the presence of surface-bound biomolecules or phytochemicals functioning as surface capping/stabilizing agents to hinder growth and improve the stability of the Ag-Cu-Co trimetallic nanoalloy particles. There are no other prominent impurity peaks observed in the EDX spectra, indicating the high-pureness of as-prepared trimetallic nanoparticles using *Salvia officinalis* extract. The elemental mapping analysis also provides concrete evidence of Ag-Cu-Co nanocomposite formation, as shown in Figure 3g. Furthermore, the elemental mapping results indicate that the Cu (Red) and Co (Yellow) are well distributed on the Ag (Green) surface (Figure 3e).

**Figure 4:**
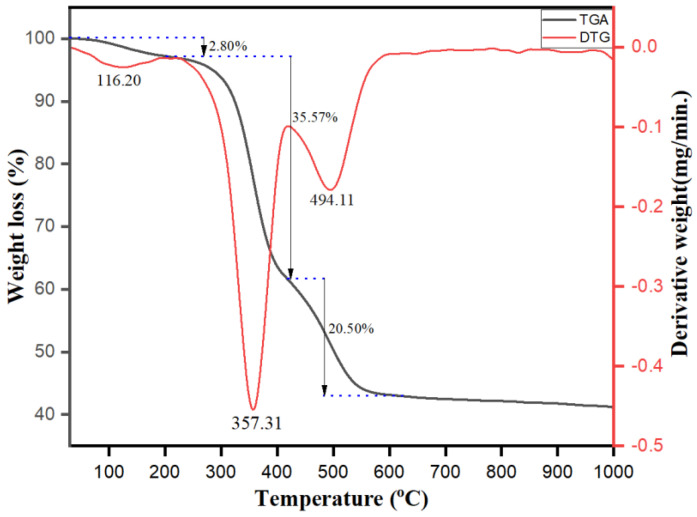
TGA-DTG curve representing thermal decomposition of Ag-Cu-Co trimetallic nanoparticles.

TGA-DTG analysis was also carried out to investigate the thermal stability and confirm the presence of surface capping phytochemicals present on the surface of the synthesized Ag-Cu-Co trimetallic nanoparticles using the aqueous extract of *Salvia officinalis* leaves as reducing and capping/stabilizing agents. The TGA-DTG curves shown in Figure 4 exhibit three-step weight loss on heating in the temperature range of 25 to 1000 °C under the nitrogen atmosphere. Our results clearly indicate that TGA curve descends until it becomes horizontal around 575 °C. Figure 4 shows that the first slight weight loss up to 220 °C is ascribed to surface adsorbed water’s physical desorption. The second and third stage rapid weight loss occurs in the temperature range of 220 °C–420 °C and 420 °C–575 °C, respectively is assigned to the thermal decomposition of the phytochemicals which are capped on the biosynthesized Ag-Cu-Co trimetallic nanoparticles. The total weight loss observed is 58.87%. The major significant weight loss between 220 °C to 575 °C makes it evident that phytochemicals present in the extract are incorporated on the surface of Ag-Cu-Co nanoparticles.

### 3.2. Anti-Candida Activity of Ag-Cu-Co Trimetallic Nanoparticle

The Ag-Cu-Co trimetallic nanoparticle was evaluated for its anti-*Candida* activity against different clinical strains of *C. auris*. The MIC values of selected strains ranged from 0.39–0.78 µg/mL, whereas MFC was twice the MIC value, ranging from 0.78–1.56 µg/mL, respectively. The MIC values for amphotericin B, fluconazole and caspofungin were found between 0.125–4.0 µg/mL, 16–500 µg/mL and 0.125–2.0 µg/mL respectively. The MIC values for nanoparticle and antifungal drugs against *C. auris* strains are enlisted in Table 2. 96% of the tested *C. auris* isolates were susceptible to caspofungin; however, it should be highly noted that only *C. auris* isolates with mutations in *FKS1* gene are considered echinocandin resistant and therefore broth microdilution results for caspofungin results should be treated as reserved [51].

In this study, we investigated the anti-*Candida* potency of Ag-Cu-Co trimetallic nanoparticles. Trimetallic nanoparticles are a more promising candidate with strong antimicrobial properties compared to their mono and bimetallic counterparts. It was recently reported that CuO-NiO-ZnO oxide and Cu-Zn-Fe oxide nanoparticles had shown promising antibacterial activity [44,52]. In another study, Au-Pt-Ag nanoparticles showed potent anti-biofilm activity against bacterial and fungal pathogenic species [12]. The nanoparticle mechanisms of action include generating reactive oxygen species (ROS), cell membrane modifications, reduction in ATP level and restricted tRNA binding to the ribosome [13]. Despite well studied, there is no concrete study reporting the anti-candidal activity of the trimetallic nanoparticles. The Ag-Cu-Co trimetallic nanoparticle at low concentration inhibits the growth of *C. auris* cells. Therefore, we intended to study further the mechanism of the antifungal action of these nanoparticles. Based on the MIC results, *C. auris* MRL6057 was selected as a representative strain for further in-depth studies.

### 3.3. Cell Count and Viability Assay

**Figure 5:**
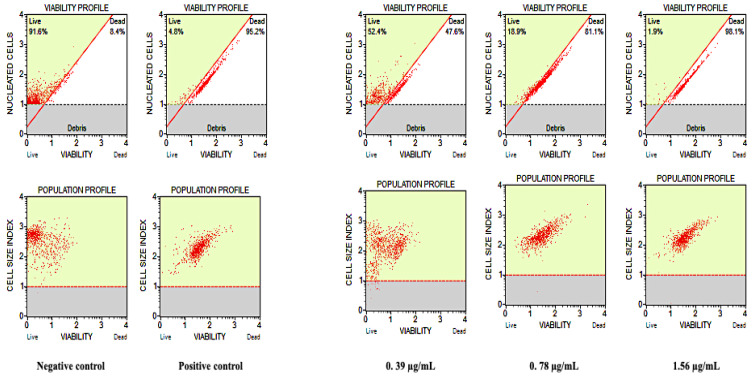
Effect of Ag-Cu-Co Trimetallic nanoparticles on cell count and viability of *C. auris* MRL 6057. The figure demonstrates the viability and population profile of *C. auris* MRL 6057. Healthy growing *C. auris* represented as negative control; cells treated with 10 mM H_2_O_2_ as a positive control; *C. auris* exposed to Ag-Cu-Co trimetallic nanoparticle at different MIC values (0.39 µg/mL, ½ MIC; 0.78 µg/mL, MIC; 1.56 µg/mL, MFC).

To further confirm the susceptibility of *C. auris* cells against Ag-Cu-Co trimetallic nanoparticles and to quantify the survival rates, cell count and viability assay were done on *C. auris* MRL6057. *C. auris* viability profile and population profile after treating with different nanoparticle concentrations are represented in Figure 5. The negative control showed healthy growing cells with 91.6% live cells, whereas, in the positive control (H_2_O_2_) only 4.8% cells were live. A dose-dependent decrease in the percentage of viable *C. auris* cells was observed after exposure to the nanoparticle. The cell viability percentage at a concentration, ½ MIC, MIC and MFC of Ag-Cu-Co Trimetallic nanoparticles was recorded as 52.4%, 18.9%, 1.9%, respectively. These results ascertained that test nanoparticle at its MFC value completely inhibits the growth and viability of *C. auris* MRL6057 and, therefore, validates anti-*Candida* potency Ag-Cu-Co trimetallic nanoparticle at its MIC and MFC values.

Due to significant antimicrobial properties, various nanoparticles have been investigated against different potential pathogenic species. Gold nanoparticles showed a wide range of biocidal activity against a broad spectrum of microorganisms [53]. Our results are incongruent with previous findings, where nanoparticles have been reported to possess high anticandidal activity [54]. The inhibitory effect of metallic nanoparticles on microbial growth and viability is a combined response of several developments, including their encounter with cell resulting in compromised membrane permeability and cell disruption, ROS formation, damage of cellular DNA and RNA, microbial cell lysis, as well as inactivation of crucial enzymes [12].

### 3.4. Apoptotic Studies

#### 3.4.1. Loss of Mitochondrial Membrane Potential (Δψm)

**Figure 6:**
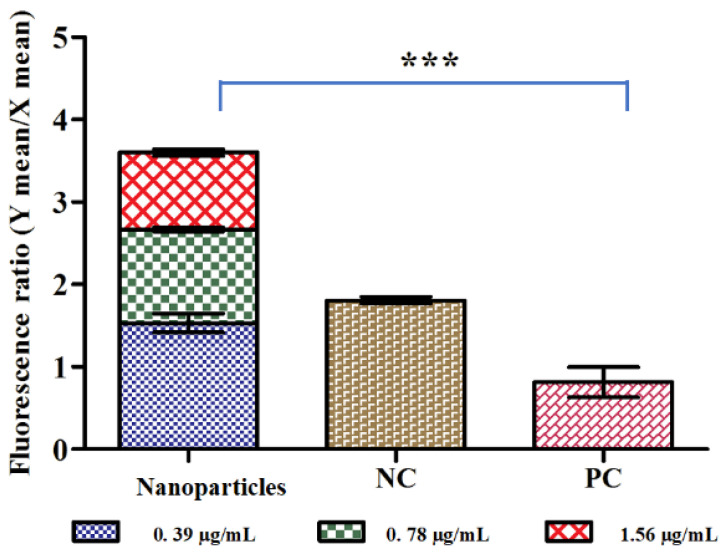
The fluorescence ratio (Y mean/X mean) as an indicator of depolarization is shown in the bar graph. Y mean represents JC-10 aggregates and X mean represents JC-10 monomers. A decrease in mitochondrial membrane potential was observed in treated *C. auris* cells compared to untreated control cells. NC: negative control; PC: positive control. *** *p* ≤ 0.05.

Mitochondria play an essential role in cell survival and apoptosis; consequently, loss of Δψm is considered a necessary step of the apoptotic pathway. Therefore, analysis of the Ag-Cu-Co trimetallic nanoparticle effect over Δψm of *C. auris* cells was crucial. Viable yeast cells have a steady Δψm and allow JC-10 dye to aggregate resulting in red fluorescence. On the other hand, apoptotic cells have lowered Δψm; the dye remains in its monomeric form giving a green fluorescence. Here, we investigated mitochondrial membrane potential in terms of the ratio of JC-10 aggregates to JC-10 monomers; a reduction in the values compared to untreated control indicated depolarization of Δψm. Compared to untreated control yeast cells, a remarkable increase in JC-10 monomer means fluorescence values have been observed, signifying the depolarization of Δψm. The ratios recorded nanoparticles exposed and unexposed yeast cells are represented in Figure 6. In the negative control, the ratio was 1.8, which lowers to 0.81 in positive control cells. In terms of nanoparticle exposure, maximum depolarization was seen at a concentration of 1.56 µg/mL (MFC) with a ratio of 0.92; whereas, at ½ MIC and MIC values, the ratios were 1.53 and 1.13 respectively, which was still lower than the negative control. These results proposed that the Ag-Cu-Co trimetallic nanoparticle tends to disintegrate the mitochondrial membrane by reducing the mitochondrial membrane potential of *C. auris* cells. Mitochondrial membrane depolarization results from unregulated mitochondrial membrane pores and therefore causes movement and triggers different pro-apoptotic factors. This feature is observed during the early stages of apoptosis and is associated with the release of *Cytochrome c*. Lemar and co-workers conducted similar work, where extracts of *Allium sativum* showed anti-*Candida* activity [55] and its constituent diallyl disulphide (DADS) showed marked mitochondrial depolarization in *C. albicans* [56].

#### 3.4.2. Ag-Cu-Co Trimetallic Nanoparticle Activates Apoptotic Factors in *C. auris*

**Figure 7:**
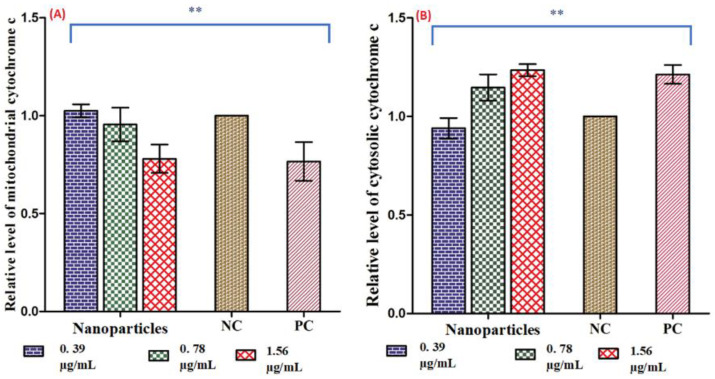
Activation of apoptotic factors in *C. auris* MRL 6057 response to Ag-Cu-Co Trimetallic nanoparticles and H_2_O_2_ (10 mM). (**A**) Mitochondrial *Cytochrome c* and (**B**) Cytosolic *Cytochrome c* was analyzed by measuring absorbance at 550 nm with a spectrophotometer. Results are based on three independent experiments and represent the average standard deviation. NC: negative control; PC: positive control. ** *p* ≤ 0.05.

The results revealed that the exposure with nanoparticle and H_2_O_2_ caused an increased cytosolic *Cytochrome c* and a decreased mitochondrial *Cytochrome c* level compared to untreated control (Figure 7A,B). A concentration-dependent release of *Cytochrome c* was recorded after exposure of *C. auris* with Ag-Cu-Co trimetallic nanoparticle. The cytosolic and mitochondrial *Cytochrome c* in untreated negative control was considered as 1.0. The average relative values in positive control cells were 1.27 and 0.76 for cytosolic and mitochondrial *Cytochrome c*, respectively. The *C. auris* cells exposed to nanoparticles MFC value showed maximum discharge of *Cytochrome c* from mitochondria, with relative values for mitochondrial cytosolic *Cytochrome c* recorded as 0.78 and 1.23, respectively.

Similarly, the relative values for mitochondrial and cytosolic *Cytochrome c* after exposure to MIC values were recorded as 0.95 and 1.14, respectively. However, at ½ MIC of nanoparticles, these values for mitochondrial and cytosolic *Cytochrome c* were almost equal to that of the negative control. Apoptotic pathways are controlled by *Cytochrome c*. It is the center for electron transfer from complex III to IV in mitochondria. Therefore, its discharge is considered a gauge for the electron transport chain [57]. Thus, these results depicted that Ag-Cu-Co trimetallic nanoparticle caused the release of *Cytochrome c* from the mitochondria and cytosol, directly affecting the electron transport chain in these cells. Hence, the nanoparticle exposure resulted in mitochondrial membrane depolarization in *C. auris* cells, followed by the bleeding of *Cytochrome c* into cytosol and finally activating yeast metacaspase Yca1p (ortholog of mammalian caspases and known to play a crucial role in yeast apoptosis). Activation of Yca1p, in turn, can trigger the caspase cascade mediated apoptosis in *C. auris*. This sequence of events is most commonly reported during apoptosis in yeast cells [58]. As noted elsewhere, hibicuslide c [59], coumarin (1,2-benzopyrone) [45] and eugenol tosylate congeners [46] also exerted anti-*Candida* activity against *C. albicans* by inducing cellular apoptosis, which was mainly due to mitochondrial dysfunction and discharging *Cytochrome c* to the cytosol. Even though these nanoparticles are structurally different from Ag-Cu-Co trimetallic nanoparticles, mitochondrial membrane depolarization results are inconsistent with these findings.

#### 3.4.3. Ag-Cu-Co Trimetallic Nanoparticles Trigger PS Externalization in *C. auris*

**Figure 8:**
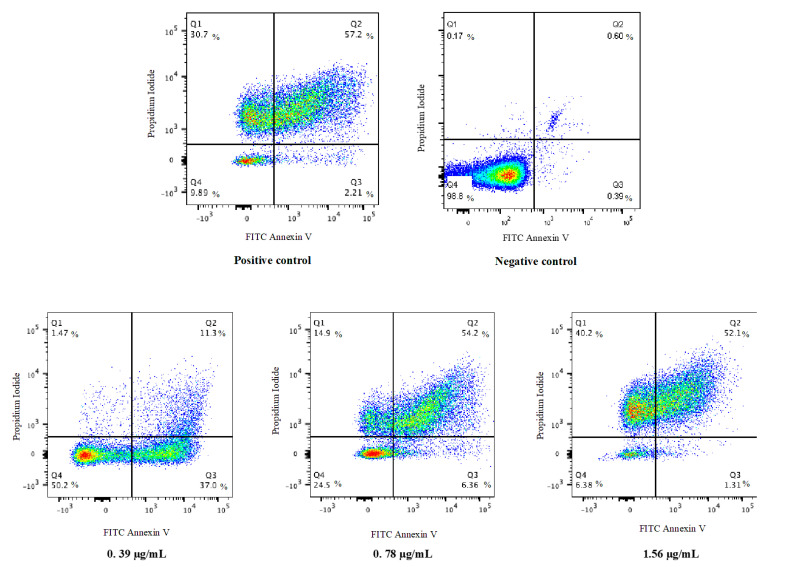
Flow cytometric analysis of phosphatidylserine exposure using AnnexinV/propidium iodide double staining. *C. auris* cells were treated with different concentrations of Ag-Cu-Co Trimetallic nanoparticles. Untreated cells were considered a negative control, whereas H_2_O_2_ (10 mM) was added for positive control. Cells exposed to Ag-Cu-Co Trimetallic nanoparticles at different concentrations values (0.39 µg/mL, ½ MIC; 0.78 µg/mL, MIC; 1.56 µg/mL, MFC) of Ag-Cu-Co Trimetallic nanoparticles.

PS externalization is the most studied apoptotic marker in yeast cells. In this study, a double staining method (Annexin V and PI) was employed to observe PS externalization in *C. auris* MRL6057 cells. The PI validates the membrane integrity of the *C. auris* cells while Annexin V stains exposed PS, allowing differentiation between apoptotic, late apoptotic and necrotic cells. After exposure of cells to ½ MIC, MIC and MFC values of Ag-Cu-Co trimetallic nanoparticle, the percentage of cells in the Q1 (Annexin V^−^/PI^+^), Q2 (Annexin V^+^/PI^+^) and Q3 (Annexin V^+^/PI^−^) quadrants has increased. In contrast, in the Q4 (Annexin V^−^/PI^−^) quadrant, the percentage of live cells has decreased significantly (Figure 8). Table 3 represents the percentage of cells present in different quadrants of the quadrant dot plot. In the negative control (untreated cells), the cell population (98.8%) was confined to Q4, representing viable cells in the sample. Whereas, in positive control cell population was distributed in all the quadrants (30.7%, Q1; 57.2%, Q2; 2.21% Q3 and 9.89%, Q4), suggesting exposure of 10 mM H_2_O_2_ mainly results in late apoptosis in *C. auris* cells. Higher concentrations of the nanoparticle resulted in a higher percentage of cells confined to Q1, Q2 and Q3. In contrast, a decrease in cell percentage was observed in Q4, revealing that the nanoparticle-induced apoptosis in *C. auris*. Furthermore, results also depicted that cells exposed to the nanoparticles sub-inhibitory concentrations showed early apoptosis. In contrast, late apoptosis was observed when cells were exposed to higher concentrations (MIC and MFC) of the Ag-Cu-Co trimetallic nanoparticle.

PS externalization is considered as an indication of early apoptosis in fungi [45]. Our results are in accordance with former findings where antifungal compounds were responsible for yeast cell membrane damage and induced apoptosis in *Candida* spp. Naphthofuranquinones compounds exhibited antifungal activity against azole-resistant *Candida* spp., as they exert toxicity by damaging the plasma membrane, depolarization of the mitochondrial membrane and DNA damage [60]. Carvacrol, a monoterpene phenol, resulted in plasma membrane depolarization and an association with apoptosis and DNA fragmentation in *C. albicans* [61]. Synthetic MCh-AMP1, a peptide, was also reported to damage the plasma membrane by increasing its permeability, induced potassium leakage and ROS production in *C. albicans* [62]. The results in this study revealed that growth inhibition of *C. auris* by Ag-Cu-Co trimetallic nanoparticle is directly linked to apoptosis.

### 3.5. Cell Cycle Arrest in C. auris

Exposure of the Ag-Cu-Co trimetallic nanoparticle-induced apoptosis in *C. auris* cells and therefore, we further studied the effect of the nanoparticle over cell cycle in *C. auris* MRL6057. Accordingly, if the nanoparticle targets gene-encoded proteins targeting cell cycle gene-encoded proteins, the percentage of cells distributed in different cell cycle phases should be altered compared to the normal growing cells, indicating cell cycle arrest. Therefore, DNA content changes were analyzed throughout different stages of the cell cycle by the Muse^TM^ Cell Analyzer, which allows the quantitative estimation of a single cell. Cells can be quantitatively differentiated in various cell cycle stages based on the fluorescence intensity produced by DNA labeled with PI, directly proportional to a particular phase in the cell cycle.

**Figure 9:**
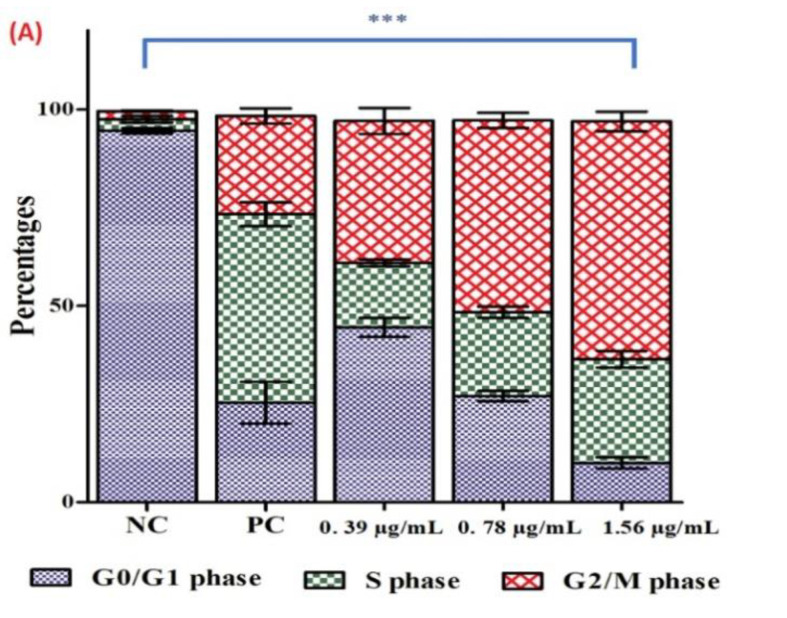

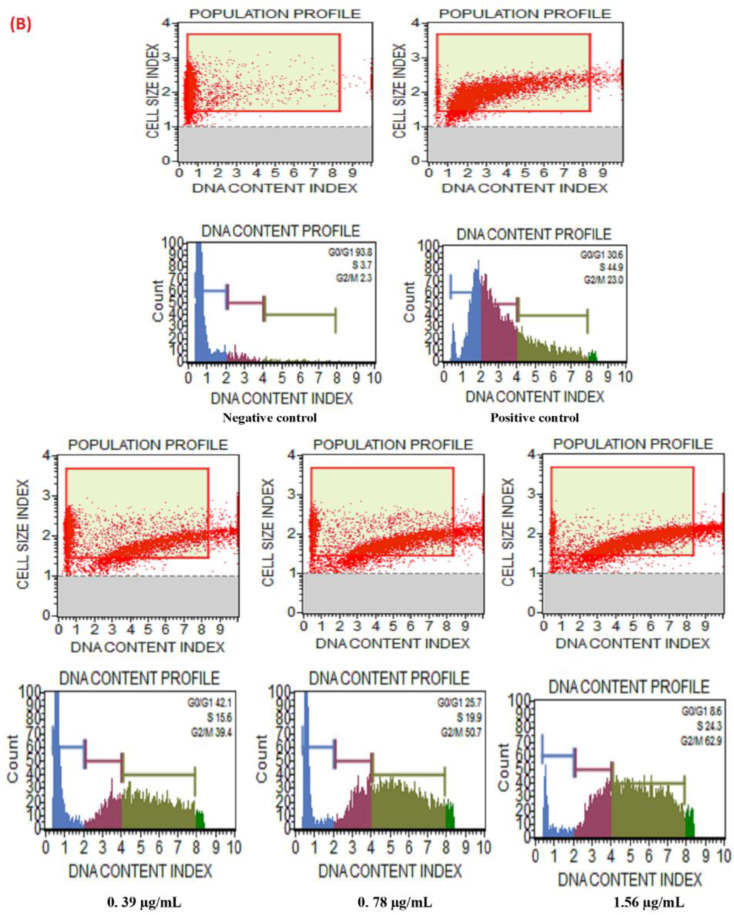
Cell cycle analysis of *C. auris* by Muse™ Cell Analyzer. (**A**) Figure showing effect of the Ag-Cu-Co trimetallic nanoparticle at ½ MIC (0.39 µg/mL), MIC (0.78 µg/mL) and MFC (1.56 µg/mL) on cell cycle progression in *C. auris* MRL 6057. Cells exposed to H_2_O_2_ (10 mM) were considered positive control, whereas untreated cells are negative control. (**B**) Representative histograms were obtained from Muse^TM^ Cell Analyzer for exposed and unexposed *C. auris* cell cycle, representing the effect of nanoparticles on cell cycle and cell size index. *** *p* ≤ 0.05.

In untreated cells (negative control), around 94.5% of cells were in G0/G1 phase, followed by 2.9% and 2.1% in S phase and G2/M phase, respectively. In positive control, 25.35%, 47.9%, 24.9% cells were recorded in G0/G1, S and G2/M phase, respectively. Exposure of *C. auris* MRL6057 cells to the Ag-Cu-Co trimetallic nanoparticle resulted in cell cycle arrest at the G2/M phase (Figure 9). Exposure of cells to sub-inhibitory concentrations of the Ag-Cu-Co trimetallic nanoparticle resulted in the accumulation of cells in G2/M phase. At a concentration of 0.39 µg/mL (½ MIC) the distribution of *C. auris* in the cell cycle was 44.5%, 16.4% and 36.1% in G0/G1, S and G2/M phase, respectively. On the other hand, at 0.78 µg/mL (MIC) of trimetallic nanoparticles, the percentage of cells in the G2/M phase increased to 48.8%, whereas the cells in S and G0/G1 phase was 21.3% and 27.0%, respectively. However, at a further higher concentration (1.56 µg/mL, MFC) the percentage of cells in G2/M phase was raised to 60.4% whereas, there was a slow increase in the percentage of cells in S phase (26.4%) and percentage of cells in G0/G1 phase decreased rapidly to 10.0%. Altogether, the results revealed that the Ag-Cu-Co trimetallic nanoparticle allowed the cells to proceed through G0/G1 and S phase, whereas it arrested the cell cycle in the G2/M phase. From these results, it is evident that the nanoparticle had a prominent effect on cell cycle progression in *C. auris*. The cells were mainly arrested in G2/M phase in a dose-dependent manner with increased arrest in G2/M phase at a higher concentration of the nanoparticle. 

Our results agree with several previous studies reporting the cell cycle arrest at the G2/M phase when *Candida* cells were treated with different external agents. Compounds, crambescidin-816, crambescidin-089, clioquinol, have been previously reported to arrest cell cycle at the G2/M phase in *Candida* spp. and *Saccharomyces cerevisiae* [63,64,65]. Impairment of cell cycle triggers changes in fungal cell morphology that increase Candida cells’ recognition by the host immune system [64]. Therefore, Ag-Cu-Co trimetallic nanoparticle directly targets *C. auris* cell cycle and enhances its recognition by the immune cells, further strengthening its candidature for anti-*Candida* treatment.

### 3.6. Haemolytic Activity of Ag-Cu-Co Trimetallic Nanoparticle

The as-prepared Ag-Cu-Co trimetallic nanoparticles showed vigorous anti-candidal activity and triggered cellular apoptosis and cell cycle arrest in *C. auris* isolates, its toxicity evaluation becomes essential. Therefore, hemolytic activity of the nanoparticle using horse erythrocytes was performed. In comparison to triton X. The percent hemolysis in the positive control was 100% whereas, there was no lysis in the negative control. In contrast to controls, Ag-Cu-Co trimetallic nanoparticle at inhibitory and sub-inhibitory concentrations was no hemolysis.

In contrast, at higher concentrations (MFC), it was observed at a rate of 0.63% hemolysis. Furthermore, at a higher concentration of 3.12 µg/mL, there was only 11.73% hemolysis. These results confirmed that Ag-Cu-Co trimetallic nanoparticle is not toxic even at 4 times higher concentration than MIC and thereby advocated the use of this nanoparticle for future in vivo studies and thereby providing a potential candidate for antifungal drug development.

The structure, size and shape determine the cytotoxicity of the nanoparticles; therefore, it is essential to enhance its stability and biocompatibility during its preparations [66]. For instance, researchers found that purification of golden nanoparticles coated with glutathione (Au-GSH NPs) by ultracentrifugation during the different steps and surface modification resulted in highly reduced cellular toxicity to human cell lines [67]. As Ag-Cu-Co trimetallic nanoparticle also undergoes several modifications during the pre-and post-synthesis processes, its cellular cytotoxicity reduced, as is evident from the hemolytic results. However, studies with human cell lines and animal models will further advocate the safe use of Ag-Cu-Co trimetallic nanoparticles.

## 4. Conclusions

The present study contributes a cost-effective and one-pot green synthesis approach to prepare stable Ag-Cu-Co trimetallic nanoparticles using *Salvia officinalis* extract as a reducing and capping agent. The phytochemicals in *Salvia officinalis* extract plays a significant role in controlling the size, morphology and size distribution of the as-prepared nanoparticles. The present study strongly supports Ag-Cu-Co trimetallic nanoparticles’ fungicidal potential against multidrug-resistant *C. auris* strains. The nanoparticles strongly impacted crucial yeast apoptotic markers as displayed by phosphatidylserine translocation and the collapse of the mitochondrial membrane potential in exposed cells. Additionally, Ag-Cu-Co trimetallic nanoparticles directly inhibit the cell cycle and arrest cells in the G2/M phase. Hemolytic studies confirmed that these nanoparticles are non-toxic and safe for stage II in vivo studies. This work indicates that Ag-Cu-Co trimetallic nanoparticles could be a potential lead for antifungal drug development. Because of their enhanced properties compared to their monometallic counterparts, trimetallic nanoparticles have emerged as useful and versatile antimicrobial nanomaterials.

## Figures and Tables

**Table 1:** The X-ray diffraction peak data of Ag-Cu-Co trimetallic nanoparticles.

S. No.	2 θ	*d*-Spacing (Å)	FWHM	Crystalline Size (nm)
1	37.7216	2.382837432	0.48178	17.42506189
2	45.47041	1.993154413	0.3593	23.97315723
3	52.86128	1.730567455	0.64428	13.76979501
4	63.97211	1.454184105	0.67217	13.93443599
5	72.52798	1.302267931	0.61254	16.08510181
6	75.6141	1.25659809	0.66303	15.16530024
7	77.90948	1.225214382	0.65923	15.49665248
8	81.72598	1.177374293	0.79933	13.14160673

**Table 2:** Minimum Inhibitory Concentrations (MIC) and Minimum Fungicidal Concentrations (MFC) of Ag-Cu-Co Trimetallic nanoparticles and three antifungal drugs representing azoles, polyenes and echinocandins against *C. auris* isolates (*n* = 25).

*C. auris* Isolates	Ag-Cu-Co Trimetallic Nanoparticle (µg/mL)		AmB * (µg/mL)	FLZ * (µg/mL)	CAS * (µg/mL)
	MIC	MFC	MIC **	MIC **	MIC **
MRL 6326	0.39	0.78	0.25 (S)	125.0 (R)	0.25 (S)
MRL 6183	0.39	0.78	0.25 (S)	250.0 (R)	0.50 (S)
MRL 4888	0.78	1.56	1.0 (S)	500.0 (R)	0.25 (S)
MRL 6015	0.39	0.78	0.25 (S)	62.0 (R)	0.50 (S)
MRL 6333	0.39	0.78	0.5 (S)	125.0 (R)	0.25 (S)
MRL 4587	0.39	0.78	0.5 (S)	32.0 (R)	0.25 (S)
MRL 6334	0.39	0.78	0.5 (S)	250.0 (R)	0.25 (S)
MRL 3785	0.39	0.78	0.125 (S)	16.0 (S)	0.25 (S)
MRL 6059	0.39	0.78	0.5 (S)	125.0 (R)	0.50 (S)
MRL 4000	0.78	1.56	2.0 (R)	250.0 (R)	0.50 (S)
MRL 6065	0.78	1.56	1.0 (S)	125.0 (R)	0.25 (S)
MRL 2921	0.78	1.56	2.0 (R)	250.0 (R)	0.25 (S)
MRL 6125	0.39	0.78	0.25 (S)	62.0 (R)	0.125 (S)
MRL 6338	0.39	0.78	0.25 (S)	125.0 (R)	0.50 (S)
MRL 3499	0.39	0.78	0.5 (S)	16.0 (S)	0.25 (S)
MRL 6194	0.39	0.78	0.25 (S)	125.0 (R)	0.50 (S)
MRL 6005	0.78	1.56	1.0 (S)	500.0 (R)	0.25 (S)
MRL 6057	0.78	1.56	4.0 (R)	125.0 (R)	2.0 (R)
MRL 5762	0.78	1.56	2.0 (R)	500.0 (R)	0.25 (S)
MRL 6173	0.39	0.78	0.25 (S)	32.0 (R)	0.25 (S)
MRL 5765	0.78	1.56	2.0 (R)	500.0 (R)	0.25 (S)
MRL 2397	0.78	1.56	1.0 (S)	16.0 (S)	0.25 (S)
MRL 5418	0.39	0.78	0.5 (S)	500.0 (R)	0.25 (S)
MRL 6277	0.39	0.78	0.5 (S)	125.0 (R)	0.25 (S)
MRL 6339	0.39	0.78	0.5 (S)	250.0 (R)	0.50 (S)

* AmB represents amphoterecin B, FLZ represents fluconazole and CAS represents caspofungin. ** (S) represents susceptible isolates and (R) represents resistant isolates using the tentative breakpoints of ≥2 (AmB); ≥32 (FLZ) and ≥2 (CAS).

**Table 3:** Percentage of cells present in different quadrants (Q1–Q4) of the quadrant dot plot. Q1: necrosis (Annexin V^−^/PI^+^), Q2: late apoptosis (Annexin V^+^/PI^+^), Q3: early apoptosis (Annexin V^+^/PI^−^) and Q4: viable cells (Annexin V^−^/PI^−^).

Condition	Quadrants	½ MIC (% Cells)	MIC (% Cells)	MFC (% Cells)
Ag-Cu-Co Trimetallic nanoparticles	Q1	1.47	14.9	40.2
	Q2	11.3	54.2	52.1
	Q3	37.0	6.36	1.31
	Q4	50.2	24.5	6.38
Positive control	Q1	30.7		
	Q2	57.2		
	Q3	2.21		
	Q4	9.89		
Negative control	Q1	0.17		
	Q2	0.6		
	Q3	0.39		
	Q4	98.8		

## Data Availability

All relevant data are within the manuscript.
